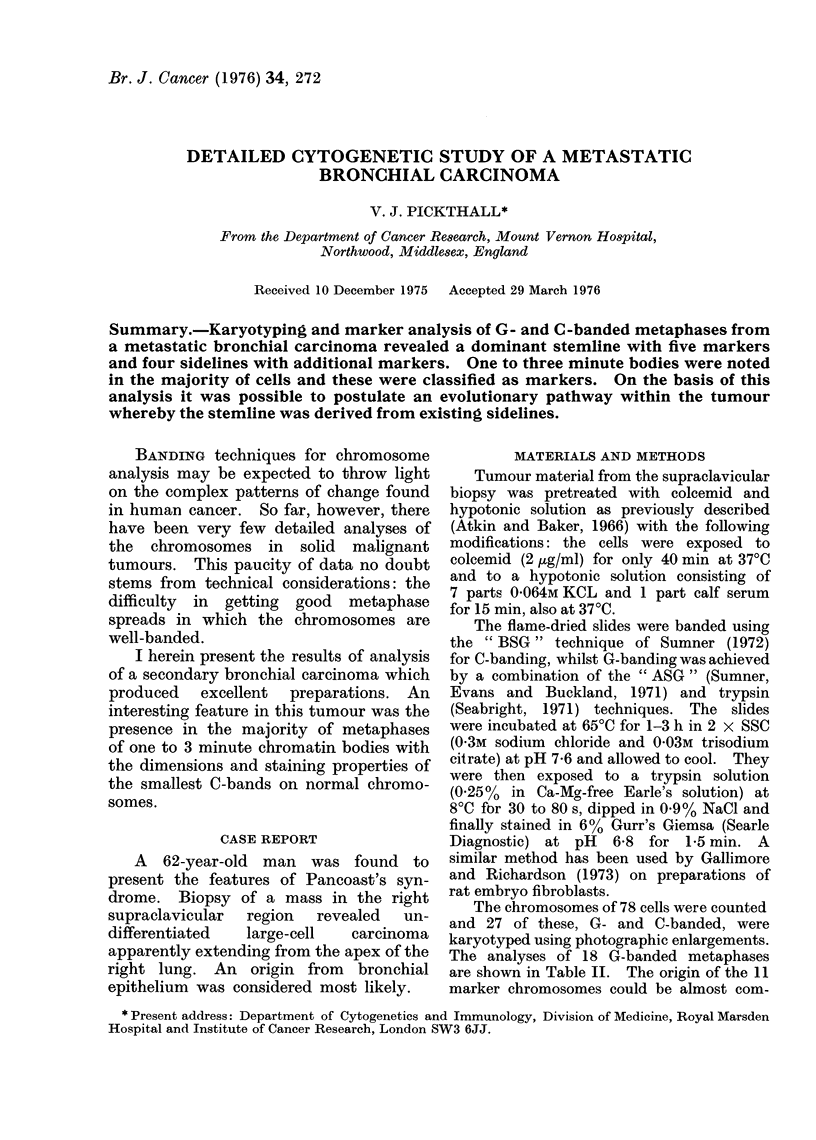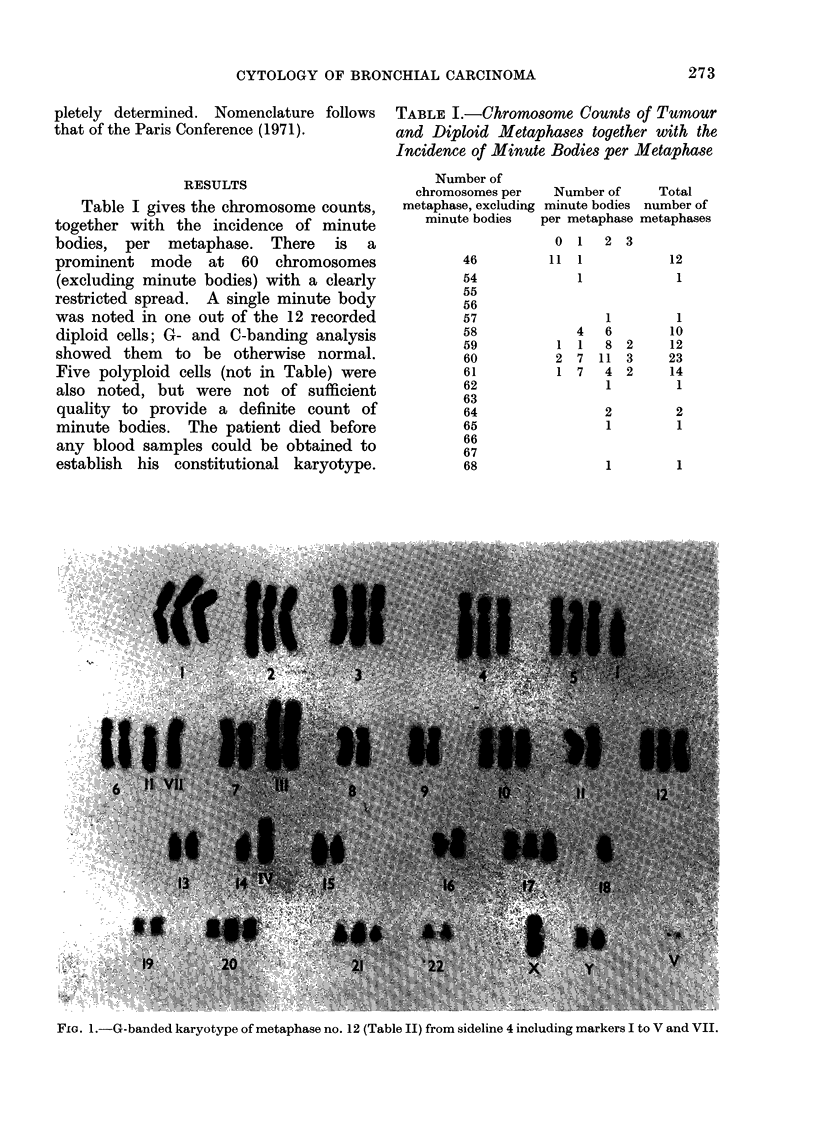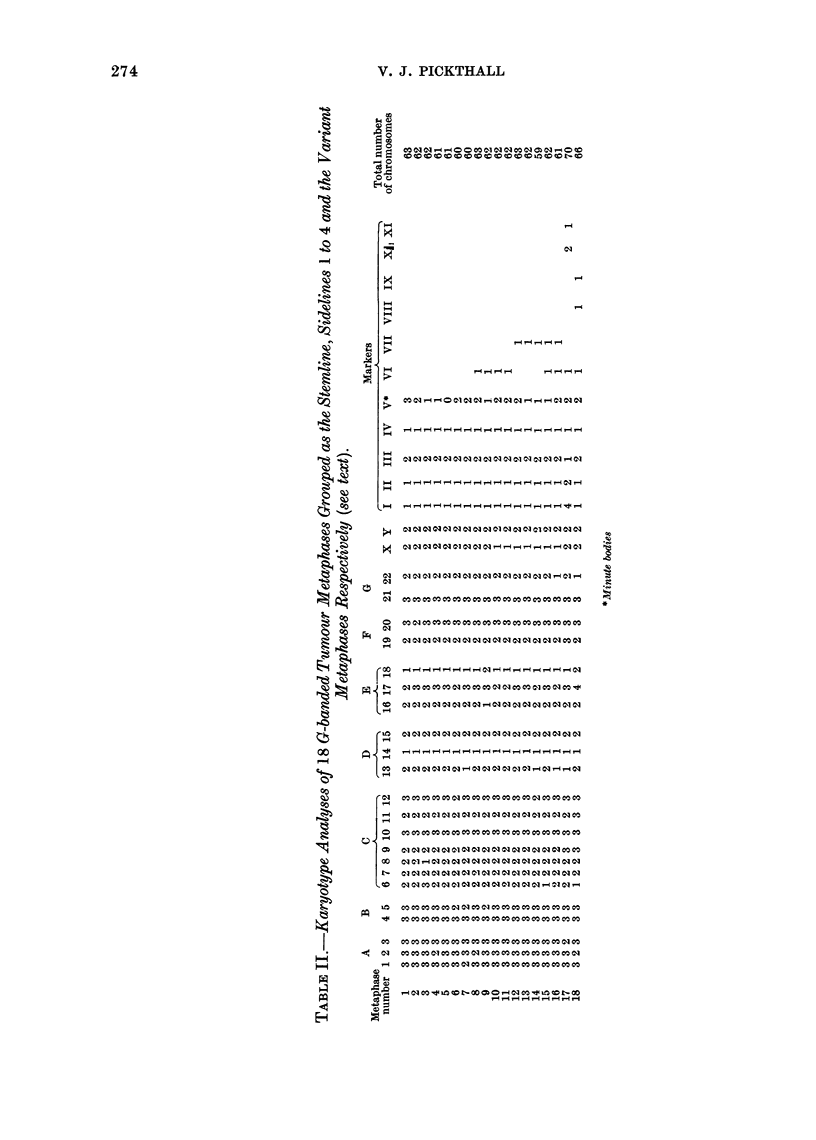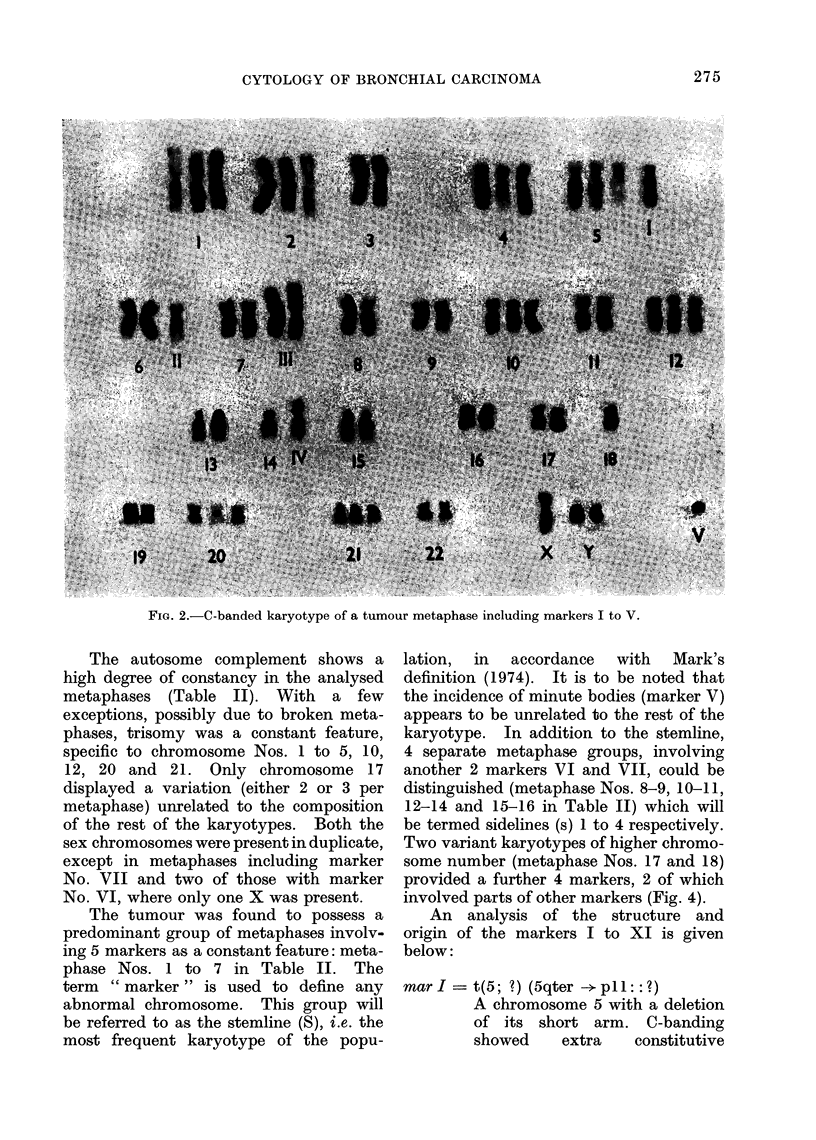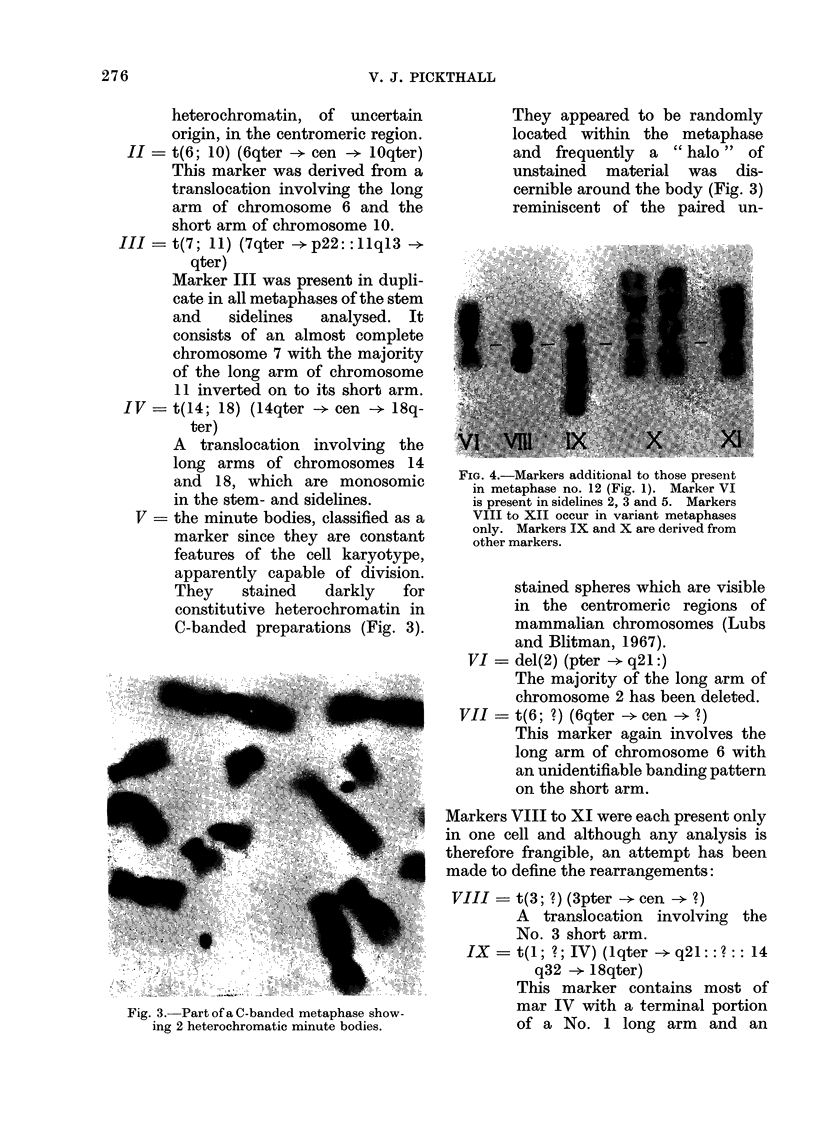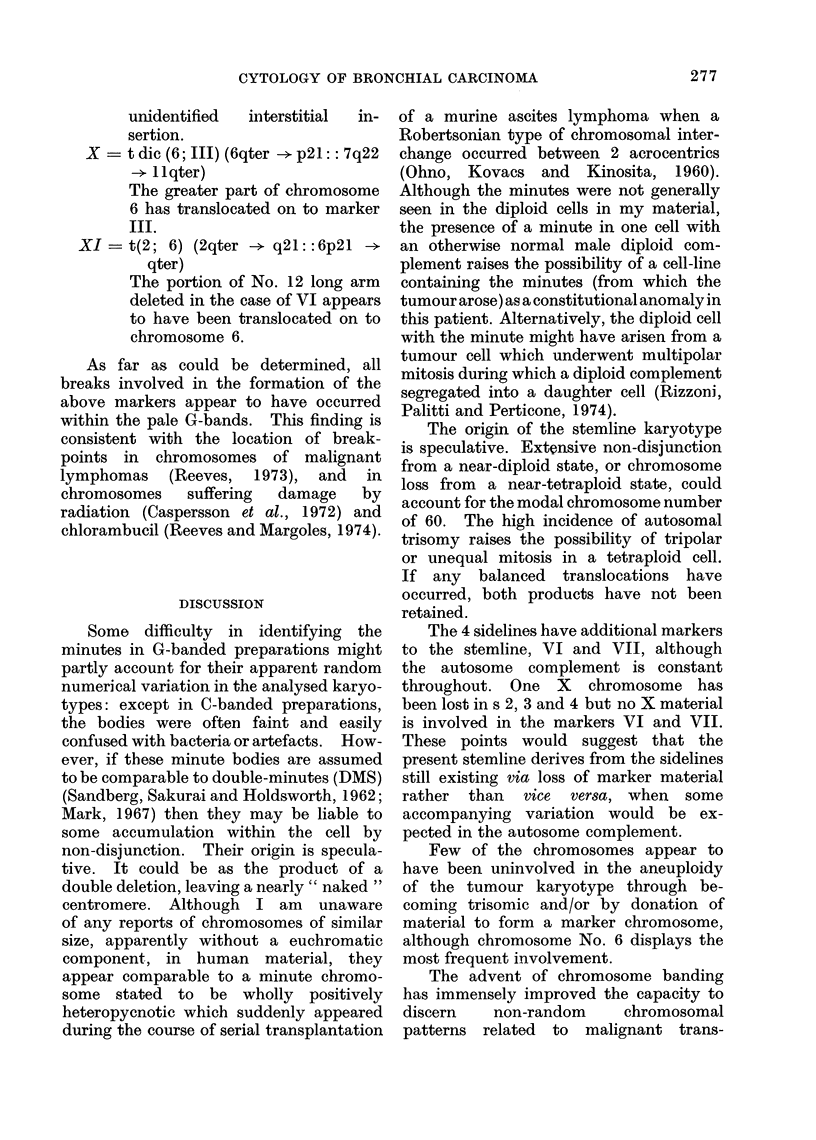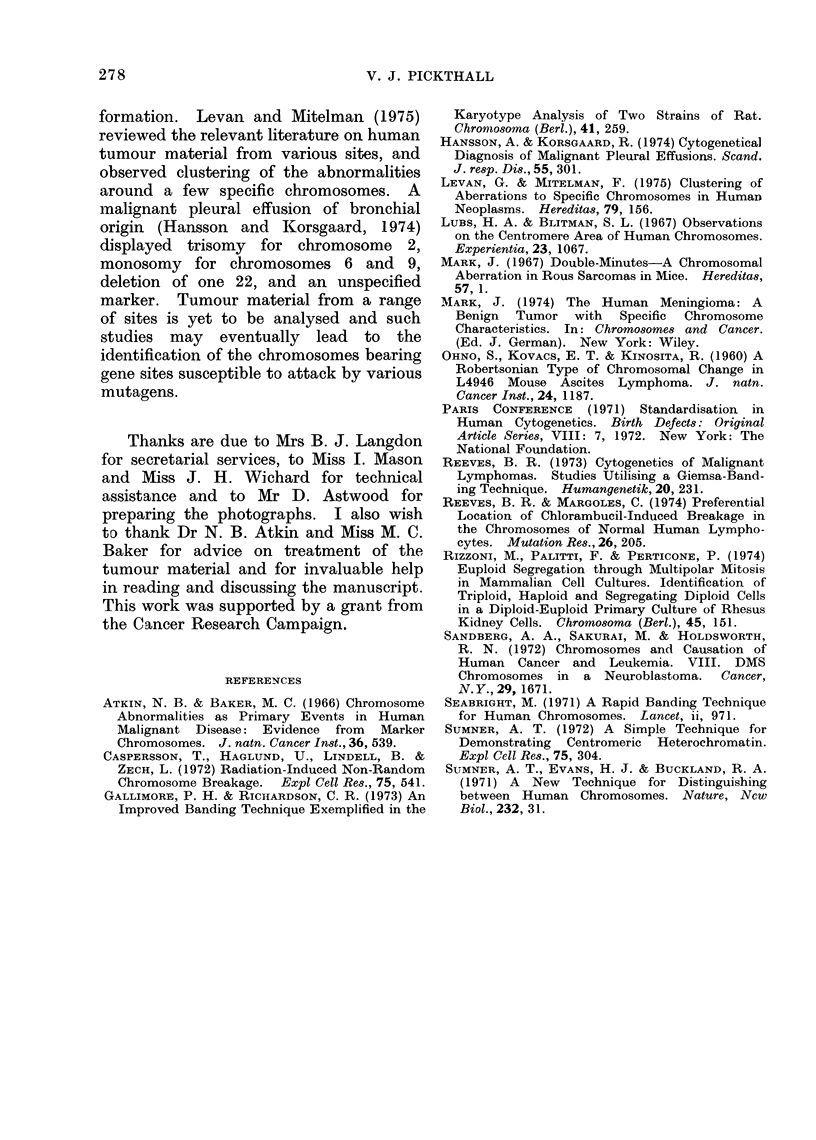# Detailed cytogenetic study of a metastatic bronchial carcinoma.

**DOI:** 10.1038/bjc.1976.162

**Published:** 1976-09

**Authors:** V. J. Pickthall

## Abstract

**Images:**


*Br. J. Cancer* (1976) **34**, 272

### DETAILED CYTOGENETIC STUDY OF A METASTATIC BRONCHIAL CARCINOMA

V. J. PICKTHALL*

*From the Department of Cancer Research, Mount Vernon Hospital, Northwood, Middlesex, England*

Received 10 December 1975 Accepted 29 March 1976

**Summary.—Karyotyping and marker analysis of G- and C-banded metaphases from a metastatic bronchial carcinoma revealed a dominant stemline with five markers and four sidelines with additional markers. One to three minute bodies were noted in the majority of cells and these were classified as markers. On the basis of this analysis it was possible to postulate an evolutionary pathway within the tumour whereby the stemline was derived from existing sidelines.**

BANDING techniques for chromosome analysis may be expected to throw light on the complex patterns of change found in human cancer. So far, however, there have been very few detailed analyses of the chromosomes in solid malignant tumours. This paucity of data no doubt stems from technical considerations: the difficulty in getting good metaphase spreads in which the chromosomes are well-banded.

I herein present the results of analysis of a secondary bronchial carcinoma which produced excellent preparations. An interesting feature in this tumour was the presence in the majority of metaphases of one to 3 minute chromatin bodies with the dimensions and staining properties of the smallest C-bands on normal chromosomes.

### CASE REPORT

A 62-year-old man was found to present the features of Pancoast's syndrome. Biopsy of a mass in the right supraclavicular region revealed undifferentiated large-cell carcinoma apparently extending from the apex of the right lung. An origin from bronchial epithelium was considered most likely.

### MATERIALS AND METHODS

Tumour material from the supraclavicular biopsy was pretreated with colcemid and hypotonic solution as previously described (Atkin and Baker, 1966) with the following modifications: the cells were exposed to colcemid (2 $\mu$g/ml) for only 40 min at 37°C and to a hypotonic solution consisting of 7 parts 0·064M KCL and 1 part calf serum for 15 min, also at 37°C.

The flame-dried slides were banded using the "BSG" technique of Sumner (1972) for C-banding, whilst G-banding was achieved by a combination of the "ASG" (Sumner, Evans and Buckland, 1971) and trypsin (Seabright, 1971) techniques. The slides were incubated at 65°C for 1–3 h in 2 × SSC (0·3M sodium chloride and 0·03M trisodium citrate) at pH 7·6 and allowed to cool. They were then exposed to a trypsin solution (0·25% in Ca-Mg-free Earle's solution) at 8°C for 30 to 80 s, dipped in 0·9% NaCl and finally stained in 6% Gurr's Giemsa (Searle Diagnostic) at pH 6·8 for 1·5 min. A similar method has been used by Gallimore and Richardson (1973) on preparations of rat embryo fibroblasts.

The chromosomes of 78 cells were counted and 27 of these, G- and C-banded, were karyotyped using photographic enlargements. The analyses of 18 G-banded metaphases are shown in Table II. The origin of the 11 marker chromosomes could be almost com-

* Present address: Department of Cytogenetics and Immunology, Division of Medicine, Royal Marsden Hospital and Institute of Cancer Research, London SW3 6JJ.

pletely determined. Nomenclature follows that of the Paris Conference (1971).

### RESULTS

Table I gives the chromosome counts, together with the incidence of minute bodies, per metaphase. There is a prominent mode at 60 chromosomes (excluding minute bodies) with a clearly restricted spread. A single minute body was noted in one out of the 12 recorded diploid cells; G- and C-banding analysis showed them to be otherwise normal. Five polyploid cells (not in Table) were also noted, but were not of sufficient quality to provide a definite count of minute bodies. The patient died before any blood samples could be obtained to establish his constitutional karyotype.

TABLE I.—*Chromosome Counts of Tumour and Diploid Metaphases together with the Incidence of Minute Bodies per Metaphase*

| Number of chromosomes per metaphase, excluding minute bodies | Number of minute bodies per metaphase | | | | Total number of metaphases |
|---|---|---|---|---|---|
| | 0 | 1 | 2 | 3 | |
| 46 | 11 | 1 | | | 12 |
| 54 | | 1 | | | 1 |
| 55 | | | | | |
| 56 | | | | | |
| 57 | | | 1 | | 1 |
| 58 | | 4 | 6 | | 10 |
| 59 | 1 | 1 | 8 | 2 | 12 |
| 60 | 2 | 7 | 11 | 3 | 23 |
| 61 | 1 | 7 | 4 | 2 | 14 |
| 62 | | | 1 | | 1 |
| 63 | | | | | |
| 64 | | | 2 | | 2 |
| 65 | | | 1 | | 1 |
| 66 | | | | | |
| 67 | | | | | |
| 68 | | | 1 | | 1 |

FIG. 1.—G-banded karyotype of metaphase no. 12 (Table II) from sideline 4 including markers I to V and VII.

TABLE II.—*Karyotype Analyses of 18 G-banded Tumour Metaphases Grouped as the Stemline, Sidelines 1 to 4 and the Variant Metaphases Respectively (see text).*

| Metaphase number | A 1 | 2 | 3 | B 4 | 5 | C 6 | 7 | 8 | 9 | 10 | 11 | 12 | D 13 | 14 | 15 | E 16 | 17 | 18 | F 19 | 20 | G 21 | 22 |
|---|---|---|---|---|---|---|---|---|---|---|---|---|---|---|---|---|---|---|---|---|---|---|
| 1 | 3 | 3 | 3 | 3 | 3 | 2 | 2 | 2 | 2 | 3 | 2 | 3 | 2 | 1 | 2 | 2 | 2 | 1 | 2 | 3 | 3 | 2 |
| 2 | 3 | 3 | 3 | 3 | 3 | 2 | 2 | 2 | 2 | 3 | 2 | 3 | 2 | 1 | 2 | 2 | 3 | 1 | 2 | 2 | 3 | 2 |
| 3 | 3 | 3 | 3 | 3 | 3 | 3 | 2 | 1 | 2 | 3 | 2 | 3 | 2 | 1 | 2 | 2 | 3 | 1 | 2 | 3 | 3 | 2 |
| 4 | 3 | 2 | 3 | 3 | 3 | 2 | 2 | 2 | 2 | 3 | 2 | 3 | 2 | 1 | 2 | 2 | 3 | 1 | 2 | 3 | 3 | 2 |
| 5 | 3 | 3 | 3 | 3 | 3 | 2 | 2 | 2 | 2 | 3 | 2 | 3 | 2 | 1 | 2 | 2 | 3 | 1 | 2 | 3 | 3 | 2 |
| 6 | 3 | 3 | 3 | 3 | 2 | 2 | 2 | 2 | 2 | 3 | 2 | 2 | 2 | 1 | 2 | 2 | 2 | 1 | 2 | 3 | 3 | 2 |
| 7 | 2 | 3 | 3 | 3 | 2 | 2 | 2 | 2 | 2 | 3 | 2 | 3 | 1 | 1 | 2 | 2 | 3 | 1 | 2 | 3 | 3 | 2 |
| 8 | 3 | 2 | 3 | 3 | 3 | 2 | 2 | 2 | 2 | 3 | 2 | 3 | 2 | 1 | 2 | 2 | 3 | 1 | 2 | 3 | 3 | 2 |
| 9 | 3 | 3 | 3 | 3 | 2 | 2 | 2 | 2 | 2 | 3 | 2 | 3 | 2 | 1 | 2 | 1 | 3 | 2 | 2 | 3 | 3 | 2 |
| 10 | 3 | 3 | 3 | 3 | 3 | 2 | 2 | 2 | 2 | 3 | 2 | 3 | 2 | 1 | 2 | 2 | 2 | 1 | 2 | 3 | 3 | 2 |
| 11 | 3 | 3 | 3 | 3 | 3 | 2 | 2 | 2 | 2 | 3 | 2 | 3 | 2 | 1 | 2 | 2 | 2 | 1 | 2 | 3 | 3 | 2 |
| 12 | 3 | 3 | 3 | 3 | 3 | 2 | 2 | 2 | 2 | 3 | 2 | 3 | 2 | 1 | 2 | 2 | 3 | 1 | 2 | 3 | 3 | 2 |
| 13 | 3 | 3 | 3 | 3 | 3 | 2 | 2 | 2 | 2 | 3 | 2 | 3 | 2 | 1 | 2 | 2 | 3 | 1 | 2 | 3 | 3 | 2 |
| 14 | 3 | 3 | 3 | 3 | 3 | 2 | 2 | 2 | 2 | 3 | 2 | 2 | 1 | 1 | 2 | 2 | 2 | 1 | 2 | 3 | 3 | 2 |
| 15 | 3 | 3 | 3 | 3 | 3 | 1 | 2 | 2 | 2 | 3 | 2 | 3 | 2 | 1 | 2 | 2 | 3 | 1 | 2 | 3 | 3 | 2 |
| 16 | 3 | 3 | 3 | 3 | 3 | 2 | 2 | 2 | 2 | 3 | 2 | 3 | 1 | 1 | 2 | 2 | 2 | 1 | 2 | 3 | 3 | 1 |
| 17 | 3 | 3 | 2 | 3 | 3 | 2 | 2 | 2 | 3 | 3 | 2 | 3 | 1 | 1 | 2 | 2 | 3 | 1 | 3 | 3 | 3 | 2 |
| 18 | 3 | 2 | 3 | 3 | 3 | 1 | 2 | 2 | 3 | 3 | 3 | 3 | 2 | 1 | 2 | 2 | 4 | 2 | 2 | 3 | 3 | 1 |

| Metaphase number | X | Y | Markers I | II | III | IV | V* | VI | VII | VIII | IX | X | XI | Total number of chromosomes |
|---|---|---|---|---|---|---|---|---|---|---|---|---|---|---|
| 1 | 2 | 2 | 1 | 1 | 2 | 1 | 3 | | | | | | | 63 |
| 2 | 2 | 2 | 1 | 1 | 2 | 1 | 2 | | | | | | | 62 |
| 3 | 2 | 2 | 1 | 1 | 2 | 1 | 1 | | | | | | | 62 |
| 4 | 2 | 2 | 1 | 1 | 2 | 1 | 1 | | | | | | | 61 |
| 5 | 2 | 2 | 1 | 1 | 2 | 1 | 0 | | | | | | | 61 |
| 6 | 2 | 2 | 1 | 1 | 2 | 1 | 2 | | | | | | | 60 |
| 7 | 2 | 2 | 1 | 1 | 2 | 1 | 2 | | | | | | | 60 |
| 8 | 2 | 2 | 1 | 1 | 2 | 1 | 2 | 1 | | | | | | 63 |
| 9 | 2 | 2 | 1 | 1 | 2 | 1 | 1 | 1 | | | | | | 62 |
| 10 | 1 | 2 | 1 | 1 | 2 | 1 | 2 | 1 | | | | | | 62 |
| 11 | 1 | 2 | 1 | 1 | 2 | 1 | 2 | 1 | | | | | | 62 |
| 12 | 1 | 2 | 1 | 1 | 2 | 1 | 2 | | 1 | | | | | 63 |
| 13 | 1 | 2 | 1 | 1 | 2 | 1 | 1 | | 1 | | | | | 62 |
| 14 | 1 | 2 | 1 | 1 | 2 | 1 | 1 | | 1 | | | | | 59 |
| 15 | 1 | 2 | 1 | 1 | 2 | 1 | 1 | 1 | 1 | | | | | 62 |
| 16 | 1 | 2 | 1 | 1 | 2 | 1 | 2 | 1 | 1 | | | | | 61 |
| 17 | 2 | 2 | 4 | 2 | 1 | 1 | 2 | 1 | | | | 2 | 1 | 70 |
| 18 | 2 | 2 | 1 | 1 | 2 | 1 | 2 | 1 | | 1 | 1 | | | 66 |

*Minute bodies

FIG. 2.—C-banded karyotype of a tumour metaphase including markers I to V.

The autosome complement shows a high degree of constancy in the analysed metaphases (Table II). With a few exceptions, possibly due to broken metaphases, trisomy was a constant feature, specific to chromosome Nos. 1 to 5, 10, 12, 20 and 21. Only chromosome 17 displayed a variation (either 2 or 3 per metaphase) unrelated to the composition of the rest of the karyotypes. Both the sex chromosomes were present in duplicate, except in metaphases including marker No. VII and two of those with marker No. VI, where only one X was present.

The tumour was found to possess a predominant group of metaphases involving 5 markers as a constant feature: metaphase Nos. 1 to 7 in Table II. The term " marker " is used to define any abnormal chromosome. This group will be referred to as the stemline (S), *i.e.* the most frequent karyotype of the population, in accordance with Mark's definition (1974). It is to be noted that the incidence of minute bodies (marker V) appears to be unrelated to the rest of the karyotype. In addition to the stemline, 4 separate metaphase groups, involving another 2 markers VI and VII, could be distinguished (metaphase Nos. 8–9, 10–11, 12–14 and 15–16 in Table II) which will be termed sidelines (s) 1 to 4 respectively. Two variant karyotypes of higher chromosome number (metaphase Nos. 17 and 18) provided a further 4 markers, 2 of which involved parts of other markers (Fig. 4).

An analysis of the structure and origin of the markers I to XI is given below:

*mar I* = t(5; ?) (5qter → p11: : ?)
A chromosome 5 with a deletion of its short arm. C-banding showed extra constitutive heterochromatin, of uncertain origin, in the centromeric region.

*II* = t(6; 10) (6qter → cen → 10qter)
This marker was derived from a translocation involving the long arm of chromosome 6 and the short arm of chromosome 10.

*III* = t(7; 11) (7qter → p22::11q13 → qter)
Marker III was present in duplicate in all metaphases of the stem and sidelines analysed. It consists of an almost complete chromosome 7 with the majority of the long arm of chromosome 11 inverted on to its short arm.

*IV* = t(14; 18) (14qter → cen → 18qter)
A translocation involving the long arms of chromosomes 14 and 18, which are monosomic in the stem- and sidelines.

*V* = the minute bodies, classified as a marker since they are constant features of the cell karyotype, apparently capable of division. They stained darkly for constitutive heterochromatin in C-banded preparations (Fig. 3).

Fig. 3.—Part of a C-banded metaphase showing 2 heterochromatic minute bodies.

They appeared to be randomly located within the metaphase and frequently a "halo" of unstained material was discernible around the body (Fig. 3) reminiscent of the paired un-

Fig. 4.—Markers additional to those present in metaphase no. 12 (Fig. 1). Marker VI is present in sidelines 2, 3 and 5. Markers VIII to XII occur in variant metaphases only. Markers IX and X are derived from other markers.

stained spheres which are visible in the centromeric regions of mammalian chromosomes (Lubs and Blitman, 1967).

*VI* = del(2) (pter → q21:)
The majority of the long arm of chromosome 2 has been deleted.

*VII* = t(6; ?) (6qter → cen → ?)
This marker again involves the long arm of chromosome 6 with an unidentifiable banding pattern on the short arm.

Markers VIII to XI were each present only in one cell and although any analysis is therefore frangible, an attempt has been made to define the rearrangements:

*VIII* = t(3; ?) (3pter → cen → ?)
A translocation involving the No. 3 short arm.

*IX* = t(1; ?; IV) (1qter → q21::?::14q32 → 18qter)
This marker contains most of mar IV with a terminal portion of a No. 1 long arm and an unidentified interstitial insertion.

$X$ = t dic (6; III) (6qter → p21: : 7q22 → 11qter)
The greater part of chromosome 6 has translocated on to marker III.

$XI$ = t(2; 6) (2qter → q21: :6p21 → qter)
The portion of No. 12 long arm deleted in the case of VI appears to have been translocated on to chromosome 6.

As far as could be determined, all breaks involved in the formation of the above markers appear to have occurred within the pale G-bands. This finding is consistent with the location of breakpoints in chromosomes of malignant lymphomas (Reeves, 1973), and in chromosomes suffering damage by radiation (Caspersson *et al.*, 1972) and chlorambucil (Reeves and Margoles, 1974).

### DISCUSSION

Some difficulty in identifying the minutes in G-banded preparations might partly account for their apparent random numerical variation in the analysed karyotypes: except in C-banded preparations, the bodies were often faint and easily confused with bacteria or artefacts. However, if these minute bodies are assumed to be comparable to double-minutes (DMS) (Sandberg, Sakurai and Holdsworth, 1962; Mark, 1967) then they may be liable to some accumulation within the cell by non-disjunction. Their origin is speculative. It could be as the product of a double deletion, leaving a nearly " naked " centromere. Although I am unaware of any reports of chromosomes of similar size, apparently without a euchromatic component, in human material, they appear comparable to a minute chromosome stated to be wholly positively heteropycnotic which suddenly appeared during the course of serial transplantation of a murine ascites lymphoma when a Robertsonian type of chromosomal interchange occurred between 2 acrocentrics (Ohno, Kovacs and Kinosita, 1960). Although the minutes were not generally seen in the diploid cells in my material, the presence of a minute in one cell with an otherwise normal male diploid complement raises the possibility of a cell-line containing the minutes (from which the tumour arose) as a constitutional anomaly in this patient. Alternatively, the diploid cell with the minute might have arisen from a tumour cell which underwent multipolar mitosis during which a diploid complement segregated into a daughter cell (Rizzoni, Palitti and Perticone, 1974).

The origin of the stemline karyotype is speculative. Extensive non-disjunction from a near-diploid state, or chromosome loss from a near-tetraploid state, could account for the modal chromosome number of 60. The high incidence of autosomal trisomy raises the possibility of tripolar or unequal mitosis in a tetraploid cell. If any balanced translocations have occurred, both products have not been retained.

The 4 sidelines have additional markers to the stemline, VI and VII, although the autosome complement is constant throughout. One X chromosome has been lost in s 2, 3 and 4 but no X material is involved in the markers VI and VII. These points would suggest that the present stemline derives from the sidelines still existing *via* loss of marker material rather than *vice versa*, when some accompanying variation would be expected in the autosome complement.

Few of the chromosomes appear to have been uninvolved in the aneuploidy of the tumour karyotype through becoming trisomic and/or by donation of material to form a marker chromosome, although chromosome No. 6 displays the most frequent involvement.

The advent of chromosome banding has immensely improved the capacity to discern non-random chromosomal patterns related to malignant transformation. Levan and Mitelman (1975) reviewed the relevant literature on human tumour material from various sites, and observed clustering of the abnormalities around a few specific chromosomes. A malignant pleural effusion of bronchial origin (Hansson and Korsgaard, 1974) displayed trisomy for chromosome 2, monosomy for chromosomes 6 and 9, deletion of one 22, and an unspecified marker. Tumour material from a range of sites is yet to be analysed and such studies may eventually lead to the identification of the chromosomes bearing gene sites susceptible to attack by various mutagens.

Thanks are due to Mrs B. J. Langdon for secretarial services, to Miss I. Mason and Miss J. H. Wichard for technical assistance and to Mr D. Astwood for preparing the photographs. I also wish to thank Dr N. B. Atkin and Miss M. C. Baker for advice on treatment of the tumour material and for invaluable help in reading and discussing the manuscript. This work was supported by a grant from the Cancer Research Campaign.

REFERENCES

ATKIN, N. B. & BAKER, M. C. (1966) Chromosome Abnormalities as Primary Events in Human Malignant Disease: Evidence from Marker Chromosomes. *J. natn. Cancer Inst.*, **36**, 539.

CASPERSSON, T., HAGLUND, U., LINDELL, B. & ZECH, L. (1972) Radiation-Induced Non-Random Chromosome Breakage. *Expl Cell Res.*, **75**, 541.

GALLIMORE, P. H. & RICHARDSON, C. R. (1973) An Improved Banding Technique Exemplified in the Karyotype Analysis of Two Strains of Rat. *Chromosoma (Berl.)*, **41**, 259.

HANSSON, A. & KORSGAARD, R. (1974) Cytogenetical Diagnosis of Malignant Pleural Effusions. *Scand. J. resp. Dis.*, **55**, 301.

LEVAN, G. & MITELMAN, F. (1975) Clustering of Aberrations to Specific Chromosomes in Human Neoplasms. *Hereditas*, **79**, 156.

LUBS, H. A. & BLITMAN, S. L. (1967) Observations on the Centromere Area of Human Chromosomes. *Experientia*, **23**, 1067.

MARK, J. (1967) Double-Minutes—A Chromosomal Aberration in Rous Sarcomas in Mice. *Hereditas*, **57**, 1.

MARK, J. (1974) The Human Meningioma: A Benign Tumor with Specific Chromosome Characteristics. In: *Chromosomes and Cancer*. (Ed. J. German). New York: Wiley.

OHNO, S., KOVACS, E. T. & KINOSITA, R. (1960) A Robertsonian Type of Chromosomal Change in L4946 Mouse Ascites Lymphoma. *J. natn. Cancer Inst.*, **24**, 1187.

PARIS CONFERENCE (1971) Standardisation in Human Cytogenetics. *Birth Defects: Original Article Series*, VIII: 7, 1972. New York: The National Foundation.

REEVES, B. R. (1973) Cytogenetics of Malignant Lymphomas. Studies Utilising a Giemsa-Banding Technique. *Humangenetik*, **20**, 231.

REEVES, B. R. & MARGOLES, C. (1974) Preferential Location of Chlorambucil-Induced Breakage in the Chromosomes of Normal Human Lymphocytes. *Mutation Res.*, **26**, 205.

RIZZONI, M., PALITTI, F. & PERTICONE, P. (1974) Euploid Segregation through Multipolar Mitosis in Mammalian Cell Cultures. Identification of Triploid, Haploid and Segregating Diploid Cells in a Diploid-Euploid Primary Culture of Rhesus Kidney Cells. *Chromosoma (Berl.)*, **45**, 151.

SANDBERG, A. A., SAKURAI, M. & HOLDSWORTH, R. N. (1972) Chromosomes and Causation of Human Cancer and Leukemia. VIII. DMS Chromosomes in a Neuroblastoma. *Cancer, N.Y.*, **29**, 1671.

SEABRIGHT, M. (1971) A Rapid Banding Technique for Human Chromosomes. *Lancet*, ii, 971.

SUMNER, A. T. (1972) A Simple Technique for Demonstrating Centromeric Heterochromatin. *Expl Cell Res.*, **75**, 304.

SUMNER, A. T., EVANS, H. J. & BUCKLAND, R. A. (1971) A New Technique for Distinguishing between Human Chromosomes. *Nature, New Biol.*, **232**, 31.